# An exploration of fibre intake and bowel function in a sample of adults at an Irish university campus

**DOI:** 10.1007/s11845-024-03764-9

**Published:** 2024-08-01

**Authors:** Ellen Lynch, Sophie Mulligan, Suzanne L. Doyle

**Affiliations:** School of Biological, Health and Sports Sciences, Technological University Dublin, Grangegorman, Dublin 7, Dublin, D07 XT95 Ireland

**Keywords:** Bowel dysfunction, Constipation, Fibre, Irritable bowel syndrome

## Abstract

**Background:**

Bowel dysfunction can significantly impair quality of life. Adequate fibre intake is associated with good bowel health but intakes have been reported to be low in Ireland.

**Aim:**

This study aimed to gather data on fibre intake and bowel habits in a cohort of adults at a university campus in Dublin, Ireland.

**Methods:**

An online questionnaire was developed by adapting validated tools to assess habitual fibre intake and bowel function. The questionnaire was circulated through mailing lists and advertised via QR codes on campus in February/March 2023. Data was analysed using SPSS, p < 0.05 was considered statistically significant.

**Results:**

In total, 275 valid responses were received. Low fibre intakes (< 19 g/day) were found in 50.5% of participants. A significantly higher proportion of males had low fibre intake compared to females (62.2% vs 44.8%, p = 0.039). Nearly a third (30.2%) of respondents experienced mild symptoms of bowel dysfunction, and 13.1% experienced moderate to severe symptoms. An inverse relationship was observed between fibre intake (g/day) and bowel dysfunction (p = 0.033).

**Conclusions:**

In this cohort, low fibre intakes and some degree of bowel dysfunction were prevalent. Public health campaigns to increase fibre intake could prove to be a cost-effective way to improve bowel function and health amongst adults in Ireland.

## Introduction

The potential protective effect of fibre against bowel disorders was first observed in the 1960s by Burkitt, who noticed the reduction of colonic diseases in Africans consuming a fibre-rich diet [1]. Gastrointestinal disturbances can manifest as constipation, diarrhoea, abdominal distension, and irritable bowel syndrome (IBS), which may be painful, potentially lowering one’s quality of life [2]. The most widely known beneficial property of fibre is its effect on gut motility and in the prevention, and treatment of constipation [3]. Other mechanisms by which fibre can benefit colonic health, independently of its effects on the microbiome, include bile acid binding, stool bulking and acceleration of colon transit [4].

The diversity of the microbiota which colonise the gut, are significantly influenced by dietary patterns [5], lifestyle habits, antibiotic usage, and disease [6]. Fibre is a key component of the diet known to modulate the composition and function of the gastrointestinal microbiota [7]. Dietary fibre refers to the edible segment of a plant which resists digestion and absorption, allowing passage into the colon, where it is completely, or partially fermented [8]. Specific species of resident colonic microbiota, which possess enzymatic machinery to break down complex carbohydrates, are responsible for fermenting fibre into short-chained fatty acids (SCFAs) [9]. The plethora of substrates fermentable dietary fibres provide can alter the microbiome and the metabolites produced [9].

Ancestral diets, consisting of fibre-rich, plant-based foods, favoured a more complex, diverse microbiome, demonstrating beneficial health outcomes [8]. In comparison, modern dietary patterns, which relate to Western diets in industrialised regions, have resulted in a considerable reduction in fibre consumption [10]. A low-fibre diet may not be sufficient to nourish the gut microbiota, leading to a loss in the species that rely on these substrates as well as a reduction in the end-products produced by fibre fermentation, which provide immunological and physiological functions [1, 11].

Constipation describes unsatisfactory defecation with infrequent, hard to pass stools and associated straining, abdominal discomfort and pain. The prevalence in the community ranges from 10–14% [12, 13]. Beyond constipation, IBS is characterised as recurrent abdominal pain associated with changes in stool form and frequency [14], with a reported global prevalence of 5–11% [15, 16]. These functional bowel disorders can significantly impact quality of life, with mental health co-morbidity often reported [12, 17] as well as significant health care utilisation [12, 18]. Whilst the pathophysiology may be multifactorial, many cases of functional bowel disorders can be managed with diet and lifestyle modification [19, 20], including increasing fibre intake.

In 2015, a randomised control trial examined the effects of fibre-rich vegetable and wholegrain powder on bowel function in constipated young adults [21]. The intervention group (n = 45) consumed 60 g/day of the powder for four weeks. Authors found the intervention significantly improved the sensation of incomplete evacuation, as well as the strain to defecate and there was a considerable increase in stool frequency (p < 0.001) compared to the control (n = 51) [21]. In 2015, a systematic review provided further evidence to support the use of dietary fibre as an effective treatment of bowel dysfunction. Authors found fibre was beneficial for chronic constipation in 5 of 7 studies reviewed, and for IBS-related constipation in 3 of 3 studies reviewed [22]. Dietary modification presents a safe, effective, and economical option for improving gut health. Indeed, it has been suggested that increasing fibre intake to alleviate constipation could reduce healthcare costs by almost €9 million in Ireland [23].

The primary aim of this study was to investigate the association between dietary fibre intake and bowel function in adults.

## Methods

This cross-sectional study investigated the association between dietary fibre intake and bowel function in Irish adults. An online survey was distributed to all campuses in Technological University Dublin for completion by staff and students. The survey went live on the 28th of February 2023 and closed after 13 days on the 13th of March 2023**.** Informed consent was obtained from all participants and ethical approval was obtained from the Research Ethics Committee and Data Protection Office in Technological University Dublin.

### Subjects

All staff and students with a registered TU Dublin email address were eligible to participate in the study. The exclusion criteria included any individuals who did not consent to participation.

### Questionnaire design

The questionnaire, created using Microsoft Forms, included questions adapted from two previously validated instruments.

Fibre intake was estimated using the dietary fibre intake short-food frequency questionnaire (DFI-FFQ) validated by Healey et al. [24]. The DFI-FFQ consists of five questions concerning the intake of high dietary fibre-containing foods (fruit, vegetables, cereals, nuts, legumes). Examples of what 1 portion of different foods amounted to, based on the food pyramid [25], were provided, with visual aids as appropriate (e.g. 1 medium carrot, 1 cup of rice). Portions were converted to gram amounts using an Irish food portion database [26] and Nutritics Version 5.87 was used to quantify the mean fibre intake from each food group assessed. Using this data, an estimation of one’s daily fibre intake could be determined. This allowed for classification into low (< 19 g fibre/day), moderate (19.01–24.99 g fibre/day), and high (> 25 g fibre/day) intake groups, with thresholds determined based on the European Food Safety Authority (EFSA) recommendations [27].

Bowel function was assessed by adapting five questions from the Initial Measurement of Patient-Reported Pelvic Floor Complaints Tool (IMPACT) (questions 1, 2, 7 and 8F) which were deemed most relevant for this study [28]. An image of the Bristol Stool Chart was included to accompany the stool-type question. An overall bowel function score ranging from 7–35 was calculated, and respondents were grouped accordingly into normal function (7–16), mild dysfunction (17–26), and moderate to severe bowel dysfunction (> 26). An additional independent question on stool frequency was included.

The questionnaire was reviewed by 8 individuals and minor edits to the interface were made based on user feedback.

### Statistical analysis

Microsoft Forms transferred the questionnaire data onto a Microsoft Excel file. Responses were coded numerically to allow for analysis. Quantitative data regarding fibre intake and bowel function were scored, totalled, and categorised into subgroups accordingly. Data were exported to the IBM Statistical Package for Social Science (SPSS) Version 28 for statistical analysis.

The normality of the distribution was tested using the Kolmogorov–Smirnov test. As this test found the data was not normally distributed, non-parametric statistical tests were used. The frequencies and percentages of participants within demographic subgroups were determined using descriptive statistics. Cross-tabulations were conducted, and the Pearson Chi-square test was used to explore the association between two categorical variables. Mann Whitney-U and Kruskal Wallis tests were used to examine the relationship between categorical and continuous variables. In all cases, p ≤ 0.05 was considered statistically significant.

## Results

### Demographic characteristics of the study population

A total of 275 valid questionnaire responses were collected. Demographic characteristics of all participants are displayed in Table 1. There was a higher response to the survey from females (69.5%) compared to males (26.9%). The majority of participants were aged between 17 and 25 years (81.5%). There was high representation from the Faculty of Science and Health with 183 members participating in the study compared to the 92 respondents from other faculties.

**Table 1:** Demographic Characteristics of the Study Population

	**n**	**%**
Total Population	275	100
Gender		
Female	191	69.5
Male	74	26.9
Non-binary	7	2.5
Prefer not to say	3	1.1
Age		
17–20	116	42.2
21–25	108	39.3
26–30	13	4.7
31–40	13	4.7
41–50	15	5.5
50 +	10	3.6
Field of Study		
Arts and Humanities	20	7.3
Business	13	4.7
Computing	14	5.1
Engineering	28	10.2
Science and Health	183	66.5
Other	17	6.2

### Fibre intake status

The reported mean daily fibre intake for the total cohort (n = 275) was 20 g (SD 11.9 g). Table 2 displays the fibre intake status of the population across demographic subgroups. Significant variances are noted in fibre intake amongst different genders and different faculties. In post-hoc analysis, when those who did not identify as male or female were removed, a significantly higher percentage of males were classed with a low-fibre status compared to females (p = 0.039). Those in the faculty of science and health were significantly more likely to have a high fibre status than those in other faculties (p < 0.001).

**Table 2:** Fibre Intake Status across Demographic Subgroups

	n (%)	Fibre Intake Status			*p*-value
		Low (< 19 g/day)	Moderate (19–24.9 g/day)	High (> 25 g/day)	
Total	275 (100)	139 (50.5)	46 (16.7)	90 (32.7)	
Gender, *n (%)*					
Female	191 (69.5)	85 (44.5)	35 (18.3)	71 (37.2)	0.034
Male	74 (26.9)	46 (62.2)	10 (13.5)	18 (24.3)	
Other	10 (3.6)	8 (80)	1 (10)	1 (10)	
Age, *n (%)*					
17–20	116 (42.2)	62 (53.4)	17 (14.7)	37 (31.9)	0.212
21–25	108 (39.3)	45 (41.7)	23 (21.3)	40 (37.0)	
26–30	13 (4.7)	11 (84.6)	1 (7.7)	1 (7.7)	
31–40	13 (4.7)	6 (46.2)	3 (23.1)	4 (30.8)	
41–50	15 (5.5)	8 (53.3)	2 (13.3)	5 (33.3)	
50 +	10 (3.6)	7 (70)	0 (0)	3 (30)	
Field of Study, *n (%)*					
Science & Health	183 (66.5)	77 (42.1)	34 (18.6)	72 (39.3)	< 0.001
Other	92 (33.5)	62 (67.4)	12 (13.0)	18 (19.6)	

Results are displayed as n (%). Crosstabulations and Pearson Chi-Square Tests were used

**Fig. 1:**
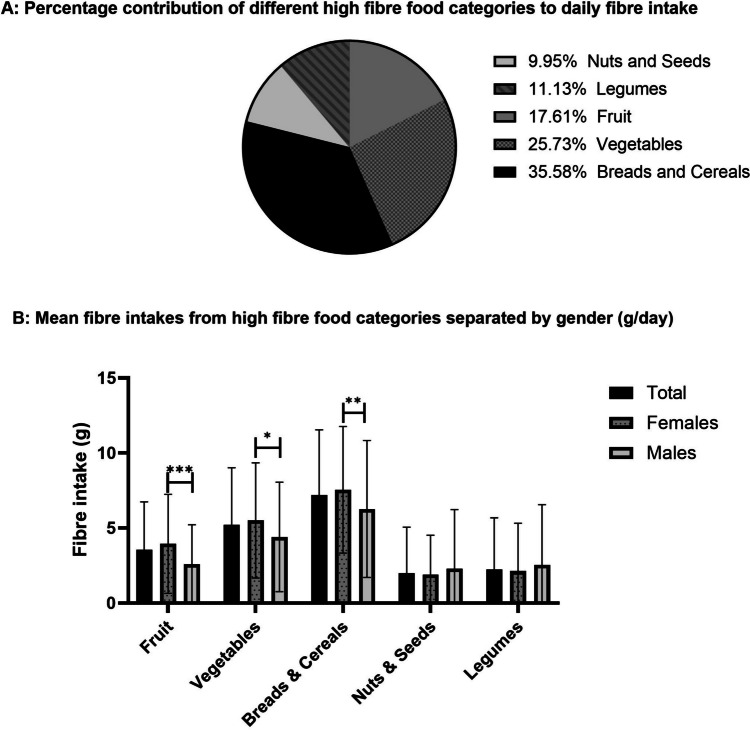
Pie chart (**A**) displaying the percentage contribution of different high fibre food categories (as per DFI-FFQ) to total daily fibre intake. Grouped bar chart (**B**) displaying mean fibre intakes in grams from high fibre food categories in the total population (n = 265), females (n = 191) and in males (n = 74). The number of portions of high fibre foods reported in the FFQ was converted to gram amounts of fibre using an Irish food portion database and Nutritics. *p < 0.005 **p < 0.01 and ***p ≤ 0.001. Error bars are displayed at a 95% CI. Mann–Whitney U tests were performed.  DFI-FFQ, Dietary fibre intake short food frequency questionnaire

The food group that contributed to most to daily fibre intake was breads and cereals (7.2 g/day), followed by vegetables (5.1 g/day) and fruits (3.5 g/day), as seen in Fig. 1A. In Fig. 1B, the mean consumption of fibre from different high fibre food groups is displayed, separated by gender. Mann–Whitney U tests revealed the mean consumption of fibre from fruits, vegetables, and breads and cereals was significantly higher in females compared to males (p = 0.001, 0.022, and 0.009, respectively).

### Bowel function status

Table 3 summarises the bowel function status of participants according to demographics. Thirty percent of participants were found to have mild symptoms of bowel dysfunction (BD), while 13.1% experienced moderate to severe symptoms. A higher percentage of males (68.9%) reported normal bowel function than females (52.4%), and similarly a lower percentage of males (9.5%) reported moderate to severe symptoms of bowel dysfunction than females (14.7%). In further analysis, excluding those who did not identify as a male or female, females had significantly higher (worse) mean bowel function scores to males (17.6 vs 15.6, p = 0.016, Mann Whitney U test). No significant differences in bowel function status were observed between age and faculty subgroups.

**Table 3:** Association between Bowel Function Status with Demographic Characteristics

Subgroup	n (%)	Bowel Function Status			*p*-value
		Normal Function	Mild BD^*^	Moderate to Severe BD^*^	
Total Population	275 (100)	156 (56.7)	83 (30.2)	36 (13.1)	
**Gender**					
Female	191 (69.5)	100 (52.4)	63 (33.0)	28 (14.7)	0.168
Male	74 (26.9)	51 (68.9)	16 (21.6)	7 (9.5)	
Other	10 (3.6)	5 (50)	4 (40)	1 (10)	
**Age**					
17–20	116 (42.2)	70 (60.3)	29 (25.0)	17 (14.7)	0.165
21–25	108 (39.3)	54 (50.0)	43 (39.8)	11 (10.2)	
26–30	13 (4.7)	6 (46.2)	4 (30.8)	3 (23.1)	
31–40	13 (4.7)	9 (69.2)	3 (23.1)	1 (7.7)	
41–50	15 (5.5)	8 (53.3)	4 (26.7)	3 (20.0)	
50 +	10 (3.6)	9 (90.0)	0 (0)	1 (10)	
**Science Faculty**					
Science & Health	183 (66.5)	103 (56.3)	58 (31.7)	22 (12.0)	0.634
Other	92 (33.5)	53 (57.6)	25 (27.2)	14 (15.3)	

^*^BD = Bowel Dysfunction. Results are displayed as n (%). Crosstabulations and Pearson Chi-Square Tests were performed

### Bowel function and fibre status

The relationship between fibre intake and stool frequency, stool type, and bowel function status is displayed in Table 4. A Pearson Chi-Square test found a significant association between fibre status and stool frequency (p = 0.008). Using a Kruskal–Wallis test, those who defecate once every 2 or 3 days were found to have significantly lower fibre intakes than those who defecate 1 or 2 times a day (p = 0.001), and those who defecate once every 2 or 3 days had significantly lower fibre intakes to those who defecate 3 or more times a day (p = 0.002).

**Table 4:** Association between Fibre Status and Stool Frequency, Stool Type and Bowel Function

Subgroup	n (%)	Fibre Intake Status			P-value
		Low (< 19 g/day)	Moderate (19–24.9 g/day)	High (> 25 g/day)	
Total Population	275 (100)	139	46	90	n/a
**Stool Frequency**					
Every 2/3 days	67 (24.4)	46 (33.1)	7 (15.2)	28 (14.7)	0.008
1–2 a day	176 (64)	80 (57.6)	35 (76.1)	61 (67.8)	
3 + a day	32 (11.6)	13 (9.4)	4 (8.7)	15 (16.7)	
**Stool Type**					
Optimal	221 (80.4)	104 (74.8)	39 (84.8)	78 (86.7)	0.148
Hard	25 (9.1)	18 (12.9)	2 (4.3)	5 (5.6)	
Loose	29 (10.5)	17 (12.2)	5 (10.9)	7 (7.8)	
**Bowel Function Status**					
Normal Function	156 (56.7)	76 (54.7)	24 (52.2)	56 (62.2)	0.440
Mild Dysfunction	83 (30.2)	41 (29.5)	15 (32.6)	27 (30.0)	
Moderate-Severe BD^*^	36 (13.1)	22 (15.8)	7 (15.2)	7 (7.8)	

^*^BD = Bowel Dysfunction. Results are displayed as n (%). Crosstabulations and Pearson Chi-Square Tests were performed

Normal stool type (type 3 or 4 on the Bristol stool chart) was present in 80.4% of participants, loose stool (type 1 or 2), was present in 10.5%, and hard stool type (type 5, 6 or 7) was present in 9.1%. When considering fibre as a continuous variable in grams, Mann–Whitney U tests revealed higher mean intakes in those with optimal stool type compared to those who had a hard stool type (20.7 g/day versus 15.9 g/day, p = 0.024). Mann–Whitney U tests also demonstrated signifcantly lower estimated mean fibre intake (g/day) in those experiencing moderate to severe symptoms of BD compared to those with normal bowel function (16.1 g/day vs 20.9 g, p = 0.033).

Table 5 summarises the relationship between stool frequency and estimated fibre intake from various sources. Significant associations were found between increased stool frequency and higher estimated intakes of fibre from fruit, vegetables, seeds and nuts, and legumes.

**Table 5:** Estimated Mean Fibre Intake from Various Sources in relation to Stool Frequency

Fruit Source (g.day)	Stool Frequency			*P*-value
	Once every 2–3 days	1–2 stools a day	3–4 stools a day	
Fruit	2.4 (2.6)	3.8 (3.2)	4.3 (3.3)	< 0.001
Vegetables	4.0 (3.3)	5.3 (3.9)	6.5 (3.9)	0.006
Breads & Cereals	6.3 (4.1)	7.4 (4.4)	7.5 (4.2)	0.179
Seeds & Nuts	1.7 (3.1)	2.0 (2.9)	2.5 (2.9)	0.017
Legumes	1.2 (1.5)	2.5 (3.7)	2.8 (4.1)	0.014

Results are presented as mean (SD), Descriptive Statistics and Kruskal Wallis Tests performed. Results do not control for total fibre intake

Additional Kruskal Wallis tests revealed a significant increase in fibre consumption from vegetables in those with optimal stool type to those with a hard stool type (5.5 g/day vs 3.0 g/day, p < 0.005). Compared to those with moderate to severe bowel dysfunction, the fibre intake from vegetables was significantly greater in those with normal bowel function (5.5 g/day vs 3.2 g/day, p < 0.001) and in those with mild bowel dysfunction (5.2 g/day vs 3.2 g/day, p = 0.007). However, total fibre intake was not controlled for in these analyses and may confound associations.

## Discussion

This present study cross-sectionally investigates the relationship between dietary fibre intake and bowel function in adults. Findings of this present study suggest the presence of a strong relationship between fibre status and stool frequency, and an association between fibre intake (g/day), and improved stool type and bowel function habits.

Assessment of fibre status revealed suboptimal intakes in this sample population. Recommendations from EFSA suggest an intake of above 25 g a day is sufficient to provide health benefits relating to optimal gastrointestinal functions [27]. Results of the present study suggest only 32.7% of the cohort met these recommendations. Authors of the National Adult Nutrition Survey [29] found 80% of participants did not meet EFSA recommendations. This suggests the 67.2% of those recorded to have suboptimal fibre intakes in the present study, may be an underestimation, possibly due to respondent bias and over-reporting of intakes of high-fibre groups. Alternatively, the reported intakes may be accurate and reflect an over-representation of health-conscious individuals given the large response rate from the faculty of health sciences. In terms of contribution from food groups, breads & cereals contributed over a third of daily fibre intake, which is aligned with the general population [29]. Gender differences were observed in terms of the amount and types of high fibre foods consumed, with females more likely to have a high fibre intake and significantly higher intakes of fibre from fruits, vegetables, and breads and cereals than males. This pattern was also observed in the National Adult Nutrition Survey [29]. It must be noted however in this cohort that intakes in both genders displayed wide standard deviations across food groups, thus the results should be interpreted with caution.

The prevalence of hard stools, indicative of constipation, was 9.1% in this cohort, which is comparable to reported community levels [12, 13]. It was observed that 72% (18/25) of those reporting a hard stool type, and 68.7% (46/67) of those defecating every 2 to 3 days or less, had a low-fibre status, thus confirming the protective effects of dietary fibre against constipation symptoms. This association endorses similar findings from an RCT conducted by Woo et al., as described earlier [21] and by other comparable studies [22]. There are many possible mechanisms which may underpin this association [3]. Insoluble fibre, found in wheat bran, vegetables, and whole grains, is beneficial for constipation as it dilutes the colon of harmful substances, absorbs undesirable colonic contents, and stimulates peristalsis, facilitating digestion [30, 31]. Gut motility is also stimulated by the short-chained fatty acids produced from fibre fermentation. SCFA are known to do this by enhancing mineral absorption and increasing the bacterial load in the colon, consequently assisting faecal transit [32]. In analyses of fibre intake by sources, significant associations were found between stool frequency and all high-fibre sources except breads and cereals, suggesting fruits, vegetables, seeds, nuts, and legumes have a greater potential to affect faecal transit. Previous studies have found fruit and vegetable fibre is more readily fermented than cereal fibre which may explain its stronger association [33, 34]. However, it must be acknowledged that the association between fibre sources and bowel frequency may be confounded by total fibre intake.

In the current study, an inverse association was present between fibre intake, and bowel dysfunction symptoms, suggesting fibre has protective effects against disordered bowel habits. These symptoms are like that of IBS, and include abdominal cramping, bloating, flatulence, diarrhoea, and constipation. Oka et al. [15] estimated the global prevalence of IBS at around 9.2% (using Rome III criteria) which is lower than the prevalence of those with reported moderate to severe symptoms of bowel dysfunction in this present study (13.1%), suggesting possible over-reporting of IBS symptoms. Oka et al. [15] also reported that woman have a higher prevalence of IBS (OR: 1.46, p < 0.001) compared to men, which corresponds with the findings in this present study. This may also account for the higher overall reporting of bowel symptoms found in this study given the higher number of female respondents. The exact pathophysiologic mechanisms of this are unknown however previous studies suggest the female sex hormones play a role [35]. Previous studies have demonstrated the positive impact soluble fibre had on the IBS symptoms, although insoluble fibre did not appear to have the same effect [36, 37]. This contradicts the findings of the present study which revealed only vegetable fibre demonstrated protective effects against disordered bowel symptoms, despite its predominant composition of insoluble fibre.

Functional bowel disorders and their management can be associated with significant healthcare utilisation [12]. Furthermore, there is strong evidence that increasing fibre intake decreases diverticular disease [38] and colorectal cancer risk [39]. In this cohort, as in the rest of the country, the majority of individuals were consuming less than the recommended 25 g/day of fibre. Thus, public health campaigns to increase dietary fibre consumption could prove to be a cost-effective, safe and efficient way to reduce disease burden associated with bowel dysfunction in Ireland.

## Strengths and limitations

The present study has several strengths. The cross-sectional design of this study allowed for data collection of considerable sample size, across a wide distribution. Validated tools were adapted, and the anonymity of the questionnaire allowed participants to honestly answer all questions concerning bowel function.

The present study is not without limitations. The cross-sectional study design can only establish an association rather than infer causality. In addition, most respondents were female, which is a common observation among previous studies involving voluntary questionnaires [40], and the majority of participants studied in the faculty of science and health. Both of these factors may influence the results. Dietary data relating to fibre may be subject to recall bias and may not reflect typical intake. The questions relating to bowel habit were adapted from a larger questionnaire, thus the validity of this shortened version has not been determined. This questionnaire relied on self-reporting of respondents which may bias the results. Data was not collected on underlying gastrointestinal disorders, and therefore residual confounding limits the strength of the study.

## Conclusion

The present study finds a significant relationship between fibre status and optimal stool type and frequency, as well as fibre intake and symptoms of bowel dysfunction. These findings suggest fibre has a protective effect against bowel dysfunction. Strategies to increase fibre intake in the Irish population should be developed to reduce the burden of bowel related disorders.

## Data Availability

Original questionnaire data is available on request from authors.
